# Assessment Precision of CT Perfusion Imaging in the Detection of Acute Ischemic Stroke: A Systematic Review and Meta-Analysis

**DOI:** 10.7759/cureus.44396

**Published:** 2023-08-30

**Authors:** Fatima Mubarak, Hareer Fatima, Muhammad Saqlain Mustafa, Muhammad Ashir Shafique, Syed Raza Abbas, Hussain Sohail Rangwala

**Affiliations:** 1 Department of Radiology, Aga Khan University Hospital, Karachi, PAK; 2 Department of Medicine, Jinnah Sindh Medical University, Karachi, PAK; 3 Department of Medicine, Dow University of Health Sciences, Karachi, PAK

**Keywords:** diagnostic accuracy analysis, imaging protocol, quadas 2 tool, transient ischemic attack, acute ischemic stroke, computed tomography perfusion, specificity, sensitivity, hemorrhagic transformation

## Abstract

Stroke, a prevalent medical emergency, comprises ischemic and hemorrhagic subtypes, with acute ischemic stroke (AIS) being a predominant type. The application of computed tomography perfusion (CTP) imaging has gained prominence due to its rapidity and accessibility in stroke evaluation. This study systematically reviews and conducts a meta-analysis of existing literature to assess the diagnostic accuracy of CTP in detecting AIS and predicting hemorrhagic transformation (HT). Employing Preferred Reporting Items for Systematic Reviews and Meta-Analyses (PRISMA) guidelines, an extensive search was conducted across electronic databases and relevant radiology journals. Studies conducted between 2007 and 2023 that fulfilled predetermined inclusion criteria underwent quality assessment using the Quality Assessment of Diagnostic Accuracy Studies 2 (QUADAS 2) tool. Cochrane diagnostic accuracy tools were used for data extraction. Thirteen studies involving a total of 1014 patients were included in the analysis. The diagnostic performance of CTP in predicting HT demonstrated high sensitivity (86.7%) and moderate specificity (77.8%), resulting in an overall accuracy of 79.1%. The negative predictive value (NPV) was notably high (92.9%), signifying its efficacy in excluding patients at risk of HT. The positive predictive value (PPV) was comparatively lower (60.3%), highlighting the need for clinical context when making thrombolysis decisions. The false positive rate was 16.2%, while the false negative rate was minimal (9.8%). Subgroup analysis underscored consistent sensitivity and specificity across diverse imaging metrics. The findings of this study emphasize the promising diagnostic accuracy of CTP imaging in predicting HT subsequent to AIS. This non-invasive technique can aid treatment decisions and patient management strategies. By effectively assessing perfusion status and offering predictive insights, CTP imaging improves stroke intervention choices, especially in identifying patients with a lower risk of HT.

## Introduction and background

Around one in four people may experience an episode of stroke in their life [1]. There are two main types of strokes: ischemic and hemorrhagic stroke. Ischemic stroke is brought on by blockages in the blood arteries that supply the brain, while hemorrhagic stroke is due to bleeding into the brain by the rupture of a blood vessel. About 60-80% of all strokes are caused by acute ischemic stroke (AIS) on average [2]. AIS is a medical emergency and is characterized by decreased blood supply to the brain, which causes brain cell destruction. Insidious onset of numbness or weakness in an arm or leg, facial droop, difficulties speaking or understanding speech, disorientation, issues with balance or coordination, and loss of vision are typical stroke signs and symptoms.

A transient ischemic attack (TIA), a transitory episode of brain dysfunction brought on by reduced blood flow, may occur before an AIS [3]. Large vascular occlusions (LVO) are responsible for 11-29% of AIS cases, which include blockages of the internal carotid artery (ICA), M1 or M2 section of the middle cerebral artery (MCA), or vertebrobasilar arteries. LVO-induced cerebral hypoperfusion can produce core infarction, the irreversible loss of brain tissue from insufficient blood flow. The penumbra is an area of hypo-perfused brain tissue surrounding the core and may be saved if quick blood flow restoration is accomplished. Recanalization in LVO instances has been successfully achieved with endovascular therapy (EVT) [4].

A frequent consequence of ischemic stroke is hemorrhagic transformation (HT), which is frequently made worse by reperfusion using alteplase (recombinant tissue plasminogen activator) or endovascular therapy (EVT). When the blood-brain barrier (BBB) is damaged, extravasation of peripheral blood into the brain results in HT. Prophylaxis is crucial because of the increased stroke morbidity and mortality caused by HT [5]. HT is 10 times more common in thrombosed patients than in those receiving placebos, regardless of the use of thrombolytic treatment, and it is linked to worse clinical outcomes [6]. More extensive hemorrhages are associated with higher risks of severe impairment and mortality, and HT is substantially connected with the results in terms of severity within three months [7].

To ensure that more patients can benefit from therapeutic intervention, doctors must be able to anticipate the probability of HT before thrombolytic intervention [8]. Because it is reasonably quick, affordable, and has few contraindications, computed tomography (CT) is a frequently utilized radiological intervention for stroke patients. Many studies have reported a relationship between magnetic resonance imaging (MRI) and CT perfusion (CTP) imaging [9-11], as well as a connection between permeability surface area measures and CTP penumbra appearances and stroke severity and HT [12,13]. The diagnostic accuracy for detecting ischemia can be increased by combining CTP with non-contrast CT [14]. In addition, CTP can also help examine the degree and reversibility of ischemia, which might help decide patients who will most likely benefit from thrombolytic therapy [15]. Sometimes CTP may not detect an ischemic lesion, resulting in a false negative assessment. Furthermore, many disorders, such as proximal intracranial stenosis and extracranial carotid artery stenosis, can imitate perfusion patterns in acute cerebral ischemia, causing a false-positive estimation [16]. Our study aims to systematically evaluate available literature to estimate the sensitivity and specificity of CTP for the detection of ischemic stroke.

## Review

Methodology

We conducted an extensive search across various electronic databases including MEDLINE (Medical Literature Analysis and Retrieval System Online), Embase (Excerpta Medica Database), Cochrane, Cumulative Index to Nursing and Allied Health Literature (CINAHL), Database of Abstracts of Reviews of Effects (DARE), and grey literature. Limited outcomes emerged from manual searches in key radiology journals. To devise our search strategy, we employed the Population, Intervention, Comparison, Outcomes and Study (PICOS) framework [17] and identified relevant terms and keywords such as 'computed tomography perfusion,' 'hemorrhagic transformation,' 'sensitivity,' 'specificity,' 'acute ischemic stroke,' and 'functional outcome,' which were subsequently utilized in each database search.

The process of selecting studies was guided by the Preferred Reporting Items for Systematic Reviews and Meta-Analyses (PRISMA) flowchart [18]. This involved initially evaluating study titles and abstracts against specific inclusion criteria, followed by a thorough examination of full texts to identify pertinent papers. Our inclusion criteria encompassed studies with a sample size of a minimum of 30 ischemic stroke patients, utilization of CTP upon admission, reference standards involving CT or MRI, reporting of diagnostic accuracy values for CTP, and studies published between 2007 and 2023. A quality assessment was performed by the Quality Assessment of Diagnostic Accuracy Studies 2 (QUADAS 2) diagnostic accuracy checklist [19].

For uniformity in data extraction, we adopted the Cochrane diagnostic accuracy data extraction tool [20]. The extracted data were subsequently subjected to a meta-analysis, a statistical technique that combines findings from multiple studies to enhance the precision and power of estimating intervention effects, reducing confidence intervals, and enhancing the identification of statistically significant effects [21]. We employed ReviewManager (RevMan) v5.4™ software (The Cochrane Collaboration, 2020) to synthesize the compiled data.

Results

**Figure 1:**
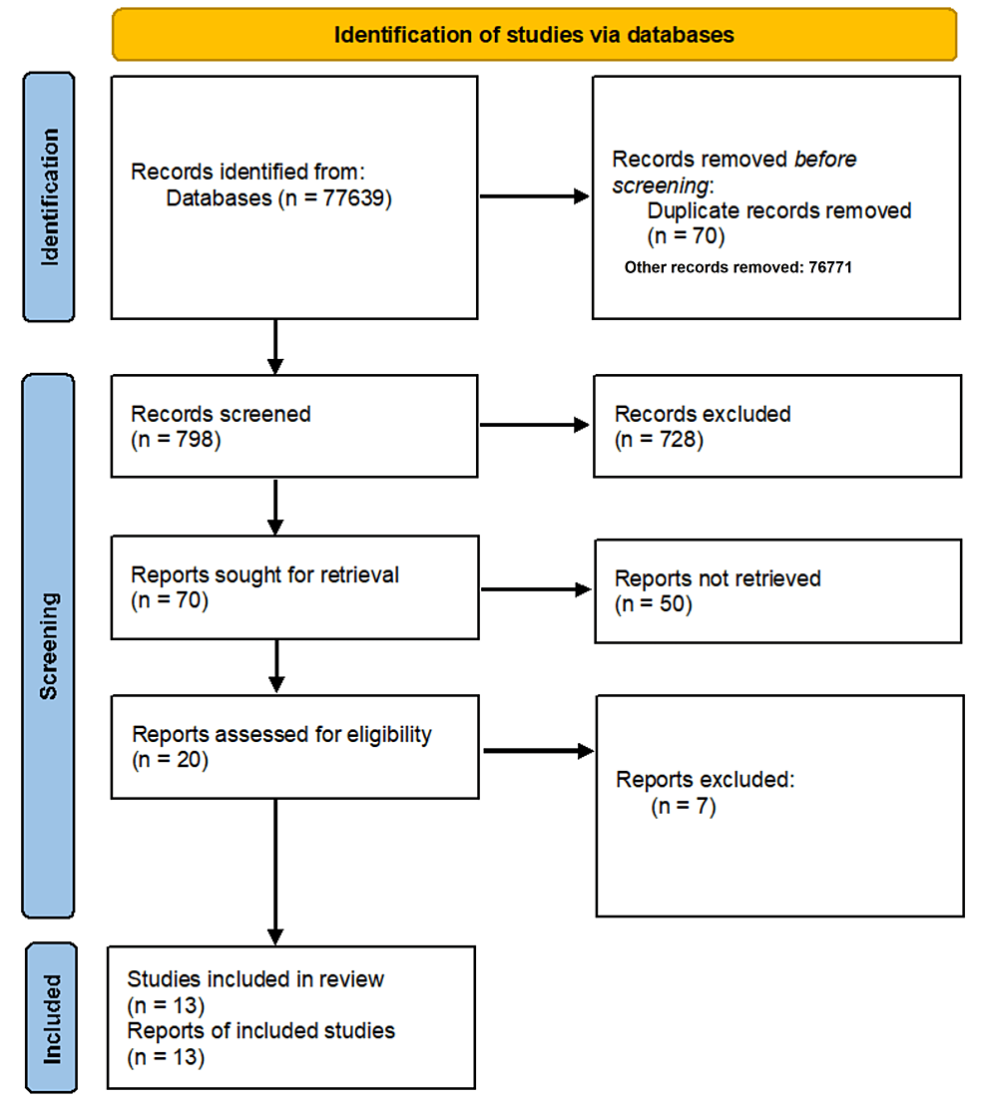
PRISMA Flow Chart PRISMA: Preferred Reporting Items for Systematic Reviews and Meta-Analyses

A total of 77,639 articles were at first sorted and narrowed down to 13 studies [22-34] based on the inclusion/exclusion criteria (Figure 1).

Quality Assessment Results

The outcomes of the assessment using the QUADAS 2 tool are displayed in Table 1. Overall, the chosen studies had high methodological quality.

**Table 1:** Risk of bias and methodological quality assessment using QUADAS 2 tool D1: Appropriate selection process?; D2: Case Control avoided?; D3: Exclusion/withdrawal explained; D4: Selection/target condition matches review question/population?; D5: Index test result blinded?; D6: Reference test result blinded?; D7; Representative index test in practice?; D8: Effect on secondary outcome considered?; D9: Reference standard correctly identify target condition?; D10: Index test reference test interval appropriate?; D11: All patients included in analysis?; D12: Pre-specified threshold used?; D13: All patients received appropriate reference standard? D14: Partial reporting/verification avoided?; D15: Diagnostic accuracy estimates/data provided?; '+'= Low Risk; '-'= High Risk; '?'= Unclear Risk QUADAS 2: Quality Assessment of Diagnostic Accuracy Studies 2

Study	D1	D2	D3	D4	D5	D6	D7	D8	D9	D10	D11	D12	D13	D14	D15
Bisdas et al. [22]	+	-	+	+	-	?	+	-	+	+	?	-	+	-	-
Hom et al. [23]	+	+	+	+	?	?	+	+	+	+	+	+	+	-	+
Yassi et al. [24]	+	+	+	+	+	+	+	-	+	+	+	+	+	+	+
Shinoyami et al. [25]	+	+	+	+	+	+	+	+	+	+	+	-	+	+	+
Bennink et al. [26]	+	-	+	+	?	-	+	-	+	+	?	-	+	-	+
Aviv et al. [27]	+	+	+	+	+	+	+	+	+	+	+	+	+	+	+
Lin et al. [28]	+	+	+	+	+	+	+	-	+	+	+	-	+	+	+
Jain et al. [29]	+	-	+	+	?	?	+	+	+	+	-	-	+	-	+
Ozkul-Wermester et al. [30]	+	+	+	+	+	+	+	+	+	+	+	+	+	+	+
Souza et al. [31]	+	+	+	+	+	+	+	-	+	+	+	?	+	+	+
Lin et al. [32]	+	+	+	+	+	+	+	-	+	+	+	-	+	+	+
Yen et al. [33]	+	+	-	+	-	?	+	-	+	+	-	-	+	-	+
Waheed et al. [34]	+	+	+	+	-	?	+	-	+	+	-	-	+	-	+

Characteristics of Included Studies

The characteristics of the 13 studies are summarized in Table 2.

**Table 2:** Characteristics of included studies. CTP: Computed Tomographic Perfusion; HT: Hemorrhagic transformation NCCT: Non-Contrast Computerized Tomography; CT: Computed Tomography; MRI: Magnetic Resonance Imaging; MRA: Magnetic Resonance Angiography

Study	Design	Year	Population	Time to CTP from onset (hours)	Reference Standard
Bisdas et al. [22]	Cohort-Retrospective	2007	68	2.5 ± 2.3	NCCT Brain
Hom et al. [23]	Cohort-Retrospective	2011	32	2 (1-12)	NCCT
Yassi et al. [24]	Cohort-Prospective	2013	132	2.5 ± 2	NCCT or MRI brain
Shinoyami et al. [25]	Cohort-Retrospective	2013	68	3 (0-24)	MRI brain
Bennink et al. [26]	Cohort-Retrospective	2015	60	1.38 (± 1)	NCCT Brain
Aviv et al. [27]	Cohort-prospective	2009	41	2.08 ± 0.77	CT and MRI
Lin et al. [28]	Cohort-Retrospective	2007	50	<3	MRI and NCCT
Jain et al. [29]	Cohort-Retrospective	2013	83 (49 analyzed)	2.07 (1.07-5.23)	NCCT or MRI
Ozkul-wernester et al. [30]	Cohort-Prospective	2014	86	2.33 (1-6)	MRI (NCCT)
Souza et al. [31]	Cohort-Retrospective	2012	96	Median 16.5 (HT) and 10.9 (No HT)	MRI (NCCT)
Lin et al. [32]	Cohort-Retrospective	2012	84	3.9 ± 2.0	MRI (NCCT)
Yen et al. [33]	Cohort-Retrospective	2016	42 (of 82 selected)	1.85	NCCT
Waheed et al. [34]	Cross Sectional-Retrospective	2023	140	<24	MRA

Patient Selection

There were a total of three studies that selected patients prospectively [24,27,30], and 10 selected them retrospectively [22,23,25,26,28,29,31-34]. The 13 studies collectively included 1014 patients, although only 948 were included in the analysis; Jain et al. [29] omitted 34 patients due to lack of matched controls, and Yen et al. [33] omitted 32 patients who had only MRI performed on follow-up. The mean age of participants was 69.9 years (33-93 years), and the median admission National Institutes of Health Stroke Scale (NIHSS) score was 11.4 (IQR 1-25).

Imaging Protocols

An average of 2.3 hours (1-24 hours) passed between the onset of symptoms and CTP. Eight studies combined the use of NCCT and MRI, whereas four studies utilized only NCCT as a reference standard. One study utilized only MRI. It took an average of 2.9 days (1-15 days) to complete the reference standard imaging. Patients who had undergone thrombolysis frequently had reference standard or confirmation imaging performed earlier.

Despite varying image capture times and methods, seven studies used measurements of the 'permeability surface (PS) area product' (PS or relative PS) for HT prediction. Souza et al. [31] suggested that the minimum time should be 120 seconds to permit enough contrast leakage into extravascular space for PS to correlate well with HT acquisition, and Lin et al. [28] asserted that increased BBB permeability is possible within two to four hours post ictus and should be visible on admission CTP performed during this time.

Since all investigations used low-osmolar non-ionic contrast media with molecular weights of 800 mg/mol 22, the diffusion performance (permeability) was comparatively consistent [35].

Thrombolysis/Thrombectomy and HT Occurrence

The 12 studies involved 361 participants (44.7%), including 134 (37.1%) who underwent thrombectomy and developed HT, and 227 who did not. However, 110 (24.6%) of the 447 patients who were not thrombolyzed went on to develop HT. So, a total of 244 patients (30.2%) experienced HT.

Diagnostic Accuracy Analysis

In 11 of the 13 studies, the diagnostic accuracy of CTP to predict HT was reported. Bisdas et al. [22] solely reported the odds ratio and p-values; two by two contingency tables were built for each study with estimated values derived from the "Mean (+/ SD)" data. According to the Centre for Reviews and Dissemination, this is a suitable strategy or method [35].

The lowest sensitivity reported was 71.4%, by Yen et al. [33], and the highest was 100%, by Hom et al. [23], Shinoyama et al. [25], and Bisdas et al. [22]. The lowest specificity was 41% [30] nd the highest was 100% [28] (Table 3).

**Table 3:** Diagnostic accuracy analysis TP: True positive; TN: True Negative; FP: False positive; FN: False negative;

Study	TP	TN	FP	FN	Sensitivity (%)	Specificity (%)
Bisdas et al. [22]	8	60	0	0	100	100
Hom et al. [23]	3	24	5	0	100	79
Yassi et al. [24]	11	80	38	3	79	68
Shinoyami et al. [25]	34	28	6	0	100	82.4
Bennink et al. [26]	16	30	10	5	75	75
Aviv et al. [27]	18	17	1	5	77	94
Lin et al. [28]	5	44	0	1	83.3	100
Jain et al. [29]	10	26	10	3	76.9	72.2
Ozkul-wernester et al. [30]	26	24	35	1	96	41
Souza et al. [31]	19	39	35	34	86.8	53.6
Lin et al. [32]	19	48	14	3	86.4	77.4
Yen et al. [33]	11	21	6	4	71.4	78.6
Waheed et al. [34]	55	4	2	40	96	91

**Figure 2:**
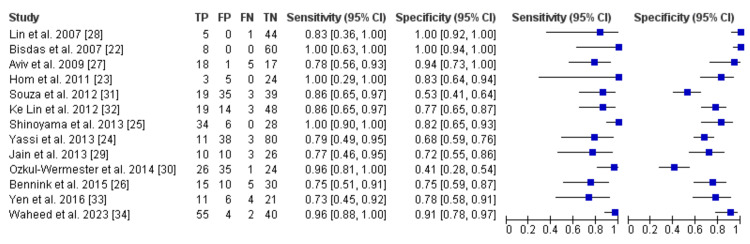
Review Manager (RevMan) v5.4 software data

**Figure 3:**
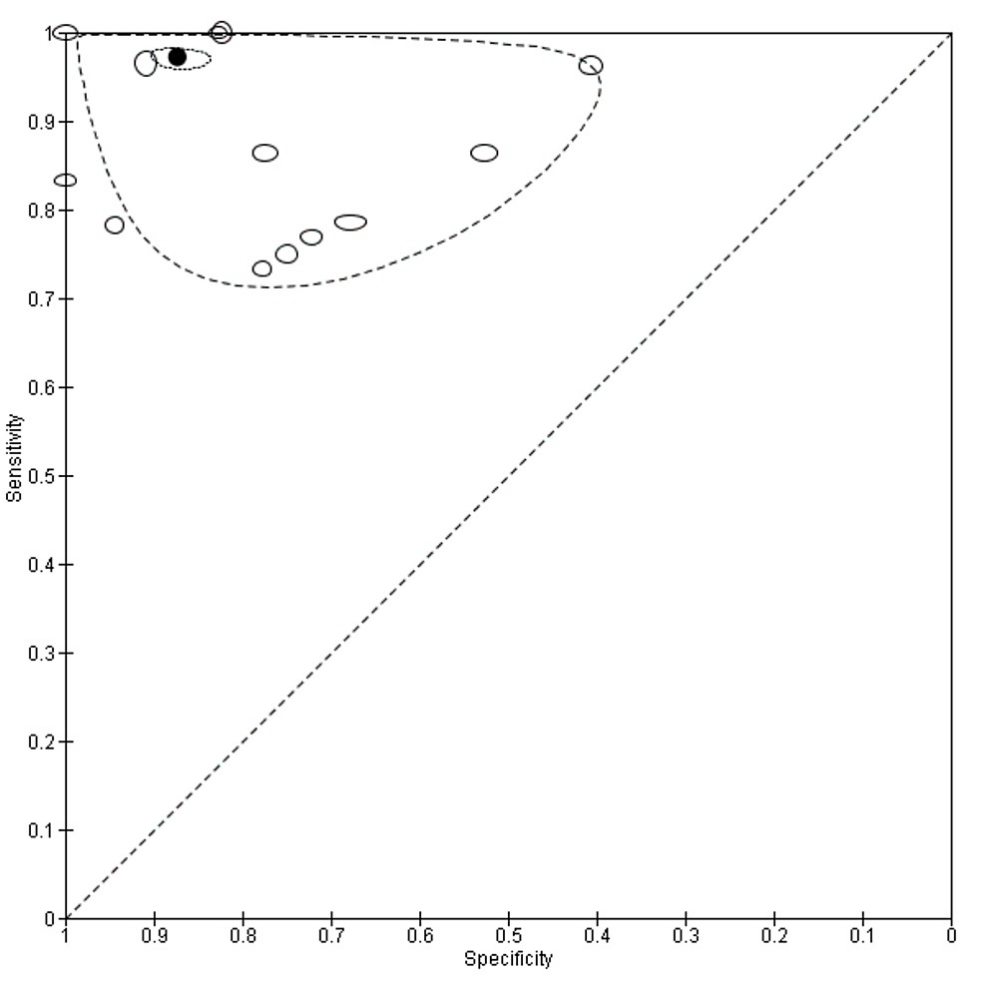
Sensitivity and specificity in ROC curve space. ROC: receiver operating characteristic

Figure 2 displays the study's sensitivity and specificity along with matched forest plots and 95% confidence intervals. The receiver operating characteristic (ROC) curve space's pooled estimations of sensitivity and specificity are displayed in Figure 3.

The total odds ratio for HT from the included studies is 2.319 (with a 95% confidence interval of 1.497-3.592 and a p-value of 0.0002) and has a 95% confidence level of 1.497. Additionally, using MedCalc Version 17.5 (MedCalc Software Ltd, Flanders, Belgium), the relative risk ratio for HT was determined to be 1.938 (with a 95% confidence interval of 1.348-2.788 and a p-value of 0.0004). The relative risk ratio indicates the relationship between a negative outcome in the experimental group (true positive) and a positive outcome (true negative) in the control group, while the odds ratio indicates the association between a negative outcome (true positive and false negative) versus a positive outcome (true negative and false positive).

Regarding whether or not recombinant tissue plasminogen activator (rtPA) was given to patients who underwent HT, the statistical analysis did not show any appreciable differences between groups of patients. In one group, 37.1% of patients experienced thrombolysis (p-value: 0.0019), compared to 24.6% in the other group (p-value: 0.0104). However, a clear distinction was found between patients who got rtPA and went on to develop HT and those who received rtPA but did not. The percentages were respectively 37.1% (p-value: 0.0019) and 62.9% (p-value: 0.998).

Discussion

The findings of this study revealed that CTP demonstrated high sensitivity (86.7) and moderately high specificity (77.8) in predicting HT, resulting in an overall accuracy of 79.1%. The high NPV of 92.9% indicates that CTP effectively identifies patients who are not at risk of HT. However, the lower PPV of 60.3% suggests that, regardless of the high sensitivity, other factors, such as the patient's condition, should also be considered when making thrombolysis decisions. A high false positive rate (16.2%; 164 out of 1014 patients) indicates that there were instances where CTP mispredicted HT in patients who did not develop it. Conversely, the false negative rate was very low (9.8%; only 99 patients), indicating that CTP was highly accurate in detecting cases where HT did occur. Overall, the power of this index test is in its ability to effectively rule out HT, making it valuable in identifying patients who are unlikely to experience this complication. However, caution is needed in interpreting positive results, and additional clinical factors should be considered in the decision-making process to optimize thrombolysis management for stroke patients.

In the analysis of 13 studies, 11 of them showed false positive results, indicating that in these studies, the test or method being evaluated incorrectly identified a condition as present when it was not present. Similarly, in 10 of the 13 studies, false negative results were demonstrated, suggesting that in these studies, the test or method being evaluated failed to identify a condition that was actually present. It's essential to interpret these findings cautiously, considering the potential reasons for false positive and false negative results, as they can impact the accuracy and reliability of the test or method under investigation [36].

Existing literature suggests that MRI has the potential to be used for predicting HT [37-39]. Various MRI perfusion maps, such as those showing extensive diffusion-weighted imaging (DWI) lesions or low apparent diffusion coefficient (ADC) [40,41], extreme hypo-perfusion (low cerebral blood flow (CBF), low cerebral blood volume (CBV), high mean transit time (MTT), prolonged Tmax) with low signal intensity on T2*W (PWI) imaging [42,43], and the presence of microbleeds on T2W-GRE or early contrast enhancement on T1W imaging, serve as useful imaging modality [42,44,45]. Nevertheless, there is disagreement regarding the particular imaging technique that best predicts HT. The current trend is toward perfusion imaging-related measurements that define a critical threshold beyond which the forecast of HT shows excellent precision and sensitivity. Examples of metrics that are currently preferred within this framework include CBV exceeding 2mL/100 g, CBF exceeding 4.9 mL/100 g/min, and MTT exceeding 145% of the opposite side [46,47].

The results of this study show that CTP imaging of the brain has a strong ability to predict HT with increased sensitivity and a reliable NPV, which can significantly increase healthcare providers' confidence in choosing stroke interventions.

Following an ischemic event, the extent of brain perfusion varies individually, and ischemia can damage the BBB, potentially resulting in HT in the most severely affected part of the brain. In the realm of clinical application, using CTP to evaluate a person's perfusion status and foresee the possible occurrence of HT gives a practical outlook to help with treatment choices. The analysis emphasizes the significant effects of both minor and substantial HT on patients' wellbeing. The combined subgroup assessment highlights the substantial sensitivity and specificity that CTP exhibits across a variety of research approaches and imaging metrics, hence reiterating its trustworthy capacity to predict HT. Healthcare professionals place a high priority on being able to accurately predict even minor cases of HT because it has a significant impact on how they decide whether to administer rtPA or alternative interventions, especially for patients who were taking other medications before their stroke. Such precise forecasts may have a significant impact on patients' results after discharge or during the 90-day follow-up period.

## Conclusions

This study demonstrates that CTP exhibits high sensitivity (86.7%) and moderately high specificity (77.8%) in predicting HT, resulting in 79.1% accuracy. A robust NPV of 92.9% suggests CTP effectively identifies HT-free patients, while a lower PPV at 60.3% indicates additional factors must complement its high sensitivity for thrombolysis decisions. The study highlights CTP's power in ruling out HT, valuable for identifying low-risk patients. Caution is advised in interpreting positive results, and clinical factors are crucial. Of 13 studies, 11 had false positives and 10 had false negatives, warranting cautious interpretation. The potential of MRI for predicting HT is acknowledged. Perfusion-related thresholds like CBV, CBF, and MTT are emerging trends for precise HT prediction. CTP brain imaging's strong predictive ability bolsters healthcare providers' confidence in stroke interventions. CTP's role in clinical application for informed treatment choices is emphasized, considering its impact on patients' outcomes.
